# Synaptic Ultrastructure Might Be Involved in HCN^1^-Related BDNF mRNA in Withdrawal-Anxiety After Ethanol Dependence

**DOI:** 10.3389/fpsyt.2018.00215

**Published:** 2018-05-29

**Authors:** Lanwei Hou, Yujuan Guo, Bo Lian, Yanyu Wang, Changjiang Li, Gang Wang, Qi Li, Jinjing Pang, Hongwei Sun, Lin Sun

**Affiliations:** ^1^Department of Clinical Medicine, Weifang Medical University, Weifang, China; ^2^Department of Bioscience and Technology, Weifang Medical University, Weifang, China; ^3^Department of Psychology, Weifang Medical University, Weifang, China; ^4^Laboratory for Cognitive Neuroscience, Weifang Medical University, Weifang, China; ^5^Department of Psychiatry and Centre for Reproduction Growth and Development, University of Hong Kong, Hong Kong, Hong Kong; ^6^Department of Rehabilitation Medicine, Han Ting People's Hospital of Weifang, Weifang, China

**Keywords:** ethanol withdrawal, anxiety, BDNF, HCN1, synaptic ultrastructure

## Abstract

Withdrawal from ethanol dependence has been associated with heightened anxiety and reduced expression of Brain-derived neurotropic factor which promotes the synaptic transmission and plasticity of synapses. Hyperpolarization-activated cyclic nucleotide-gated channel 1 regulates expression; however, whether Hyperpolarization-activated cyclic nucleotide-gated channel 1-related Brain-derived neurotropic factor is involved in the synaptic ultrastructure that generates withdrawal-anxiety has been poorly perceived. Sprague–Dawley rats were treated with ethanol 3–9% (v/v) for a period of 21 days. Conditioned place preference and body weight were investigated during ethanol administration. Rats were subjected to behavioral testing and biochemical assessments after ethanol withdrawal, which was induced by abrupt discontinuation of the treatment. The results showed that the ethanol administration induced severe ethanol dependence behaviors, with higher body weight and more time in the ethanol-paired compartment. After withdrawal, rats had a higher total ethanol withdrawal score and explored less. Additionally, increased Hyperpolarization-activated cyclic nucleotide-gated channel 1 protein and gene expression and decreased Brain-derived neurotropic factor protein and gene expression were detected in the Ethanol group. Eventually, there was a negative correlation between the level of Brain-derived neurotropic factor mRNA and Hyperpolarization-activated cyclic nucleotide-gated channel 1 protein. Importantly, the synaptic ultrastructure changed in the Ethanol group, including increased synaptic cleft width and reduction in postsynaptic density thickness or synaptic curvature. The synthesis of the Brain-derived neurotropic factor mRNA could be down-regulated by higher Hyperpolarization-activated cyclic nucleotide-gated channel 1 protein expression. Changes in synaptic ultrastructure may be induced by lower Brain-derived neurotropic factor protein, which could be associated with the withdrawal-anxiety that is experiences after ethanol dependence.

## Introduction

Alcohol dependence is chronic relapsing disorder characterized by repeated episodes of withdrawal and then relapse with resumption of heavy alcohol consumption (1). Abrupt cessation of regular alcohol intake in a dependent person causes withdrawal syndrome that is characterized by tremors, increased risk of convulsions, and anxiety (2). Ethanol withdrawal-anxiety syndrome refers to symptoms that have been shown to depend on molecular and cellular adaptations that lead to persistent, long-term plastic changes in transcription, translation, and synaptic morphology (3). However, the molecular mechanism underlying the anxiogenic effects of ethanol withdrawal has not yet been completely elucidated.

Previous studies found that changes in the synaptic ultrastructure and morphology in the nucleus accumbens (NAc) and hippocampus (Hip) are involved in various behavioral sequelae, including movement control, motivation, and addiction (4, 5). Ethanol withdrawal is accompanied by molecular and cellular adaptations that lead to persistent, long-term plastic changes in transcription, translation, and synaptic morphology (3, 6). In particular, withdrawal syndrome after drug administration is manifested by the induction of rapid changes in synaptic ultrastructure and morphology (7). However, there have been few if any reports describing whether an association exists between withdrawal anxiety and changes in synaptic ultrastructure.

Brain-derived neurotrophic factor (BDNF), a neuromodulator in the mammalian nervous system, seems to contribute to the neuroadaptive changes set in motion by drug withdrawal (8). Abstinence from chronic ethanol exposure also affects the expression of BDNF in the dentate gyrus and CA3 region of the Hip (9). Additionally, the production of BDNF mediates the anxiety induced by drug withdrawal (10). Importantly, BDNF plays a crucial role in regulating synaptic transmission and plasticity at adult synapses. A lower level of BDNF in all brain structures could lead to an increase in the synaptic cleft width and reduction in postsynaptic density thickness (11).

It has been found that BDNF could be up- regulated by a lentivirus-based silencing RNA system using short hairpin RNA (shRNA)-hyperpolarization-activated cyclic nucleotide-gated channel 1 (HCN1), which reduced the expression of HCN1 protein, increased the neuronal excitability, and produced anxiety- and depressive-like behavior (12). Studies conjectured that hyperpolarizing membrane was generated by Ih current when HCN1 channel excessively activated, which inhibited calcium inward current of the presynaptic membrane. And the BDNF released from presynaptic vesicles into the synaptic cleft, dependent intracellular calcium concentration, were prevented in the neuron which might lead to the anxiety (13). Additionally, HCN1-related BDNF played a crucial role in the pathogenesis of anxiety were explored in our previous study which found there was a negative correlation between the level of BDNF and HCN1 in the brain (14).

HCN1 is known to play a fundamental role in controlling several important cellular functions including dendritic integration, synaptic transmission, plasticity phenomena, and rhythmic activity in the central nervous system (15, 16). Additionally, HCN1 is expressed at the highest levels in the NAc and Hip, which are the most important brain regions involved in the process of drug addiction (17, 18). One *in vitro* study reported that withdrawal from repeated methamphetamine administration increased HCN1 mRNA levels (19). Repeated ethanol exposure *in vivo* down-regulated Ih in the HCN1 (20). Although Mala observed that inactive HCN1 channels might be enhanced when the BDNF protein releases from presynaptic vesicles into the synaptic cleft (13), the molecular mechanisms by which reduced HCN channel function augmented BDNF are still not clearly understood. Additionally, the potential effect of HCN1 in ethanol withdrawal has not been reported.

Based on this background, the present study hypothesized that changes in synaptic ultrastructure, which could be induced by a reduction of BDNF due to an increase in HCN1 expression in the Hip and NAc, might be associated with ethanol withdrawal-induced anxiety. An attempt was carried out to further understand this potential mechanism regarding ethanol withdrawal-induced anxiety. This study was conducted to establish an ethanol-dependent rat model (21). After ethanol withdrawal, we examined the withdrawal syndrome using behavioral tests, as well as variations in the expression of BDNF and HCN1 proteins in the prefrontal cortex (PFC) by biochemical tests. Additionally, the synaptic ultrastructure and morphology were observed by transmission electron microscopy (TEM).

## Materials and methods

### Animals

The male Sprague–Dawley rats (6 weeks old, 180 ± 20 g) utilized in this study were purchased from the animal center at WeiFang Medical University. The animals were housed five per cage (53.5 × 39 × 20 cm) with a 12-h light/12-h dark cycle (lights on at 06:00 a.m.) at a relatively constant room temperature (23 ± 1°C) and humidity (45%). Food was available *ad libitum*. Sterilized drinking water or ethanol solutions were available *ad libitum*, with the exception of the periods determined for withdrawal as follows. Before the experiments, the rats were undisturbed for 7 days to acclimate to the environmental conditions. All experiments were performed between 9:00 a.m. and 14:00 p.m. during the light cycle. The experiments were conducted according to the National Institutes of Health Guidelines (Care and Use of Laboratory Animals) and were approved by the WeiFang Medical University Animal Care and Use Committee.

### Ethanol administration

**Figure 1:**
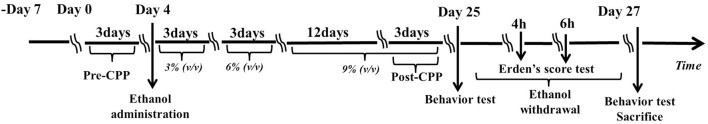
Experimental schedule for developing the ethanol dependence and withdrawal model. On day 0, rats were identified any pre-existing bias toward the individual compartments in the pre-conditioning test for 3 days. On day 4, the rats were administrated a solution of 3% ethanol (v/v) that was increased gradually every 3 days to 6% (day 7–9) and to 9% (day 10–24). The post-conditioning test was developed on the days 22, 23, and 24. The Erden's score was tested for 4 min on hours 4 and 6 after ethanol withdrawal. Then, behavioral assessments, including the open field test (OFT) and the elevated plus maze (EPM) test were administered on day 25 and day 27. All the rats were sacrificed immediately after behavioral assessments on day 27.

The control group (*n* = 20) animals received water *ad libitum* for 23 days. The ethanol group (*n* = 20) received ethanol starting with a solution of 3% ethanol (v/v) that was gradually increased every 3 days to 6% (days 7–9) and then 9% (days 10–24). Then, the ethanol solution (9%) was removed and returned the next day (day 24) for 2 h. After that, the animals received water until day 26, thereby ensuring a 48 h abstinence period. Previous studies showed that anxiety-like behavior after this period of abstinence from ethanol was robust (22). Therefore, a period of 48 h after ethanol withdrawal was chosen in this study. A short period of treatment using a low dose of ethanol was chosen to avoid any systemic or vascular effects of the ethanol. The choice of treatment for 21 days with ethanol (3–9%) was based on a previous study (21) (See Figure 1).

### Determination of the effect of ethanol

#### Body weight

All animals were weighed once daily throughout the 21 days of ethanol treatment.

#### Conditioned place preference

The reward effect was assessed by conditioned place preference (CPP) testing with a two-compartment CPP apparatus (23). In brief, the apparatus consisted of two wooden chambers identical in size (30 × 30 × 30 cm). The first chamber had white walls with a large textured grid floor, whereas the second chamber was black with a smooth floor, and they were connected to the other two compartments via removable doors. Both black and white compartments had a ceiling lamp. General activity was monitored via a video recorder for 15 min, and the time spent in each compartment was recorded using Smart3.0 software (Version 3.0, Panlab SL, Barcelona, Spain). CPP consisted of a 3-day schedule with two distinct phases: a pre-conditioning test (pre-CPP) on Day 1–3 and a post-conditioning test (post-CPP) on Day 22–24.

To identify any pre-existing bias toward the individual compartments, each rat was initially placed in the middle chamber and allowed to freely explore the entire apparatus for a 15-min session for three consecutive days before ethanol administration. The time spent in each compartment on the third day was used as the pre-conditioning data. The most preferred compartment was designated as the preferred compartment, and the other as the non-preference compartment. Each animal received individual CPP training. In the post-CPP session, each rat was allowed to freely explore the entire apparatus for a 15-min session repeatedly on Day 22–24 in an ethanol-free state. The amount of time spent in the non-preference (ethanol-paired) compartment was recorded as the post-conditioning data.

### Ethanol withdrawal symptoms

#### Erden's score

The symptoms of ethanol withdrawal were tested for 4 min 4 and 6 h after ethanol withdrawal. At each observation time, the rats were simultaneously assessed for the following behavioral conditions: stereotyped behaviors (grooming, sniffing, head weaving, gnawing, and chewing), agitation, tail stiffness, abnormal posturing, and abnormal gait. These behaviors were measured in the open-field test (OFT) (40 × 40 × 35 cm). Before the start of the experiment, the rats were put in the open field arena for 30 s. The symptoms of ethanol withdrawal were scored using a rating scale prepared by Erden (24). The behavior scores were recorded and summed for the individual observation period.

#### Open-field test

Exploratory locomotor activity and anxiety were tested in the OFT as previously reported (25). The area consisted of an enclosed square arena (100 × 100 × 50 cm). The field was divided into 25 squares with computer virtual grid lines for analysis using Smart3.0 software. Each rat was placed in the central area and was allowed to explore for 5 min. Their behaviors, including the number of crossings (with all four paws placed in a new square), upright posture (both front paws raised from the floor), carding, and fecal grains were recorded by a digital camera.

#### Elevated plus-maze test

The standard EPM test was used to assess anxiety-like behavior (26). The EPM apparatus was composed of two open arms (50 × 10 cm) and two closed arms (50 × 10 × 40 cm) connected by a central platform (10 × 10 cm). The EPM was made of dark gray plastic and positioned 100 cm above the floor. The rats were individually placed in the central platform, facing the closed arms, and allowed to explore the arena freely for 5 min. The behaviors, including the number of entries into the open and closed arms, as well as the percentage of time spent in the open and closed arms were recorded by a digital camera.

### Immunohistochemistry

Immunohistochemistry was performed as previously described (27). The rats were deeply anesthetized and transcardially perfused with normal saline followed by paraformaldehyde in phosphate buffer. Then, coronal cryotome sections (20 μm) were cut through the Hip and NAc using a cryostat and collected on poly-L-lysine-coated slides. After drying overnight at room temperature, the sections were treated with 0.3% H_2_O_2_ in methanol. After rinsing in phosphate-buffered saline (PBS) three times, the sections were blocked in 5% normal goat serum at room temperature. Next, the brain sections were incubated overnight at 4°C in primary antibody (anti-HCN1, 1:500, ab84816; anti-BDNF, 1:500, ab108319; both from Abcam, Cambridge, MA, USA) solutions with Primary Antibody Dilution Buffer. After repeated washing, the brain sections were incubated at room temperature with secondary antibody (Peroxidase-Conjugated Affinipure Goat Anti-mouse IgG (H+L), 1:200 dilution, ZB-2305, ZSGB-BIO, 1:200 dilution; Peroxidase-Conjugated Affinipure Goat Anti-Rabbit IgG (H+L), 1:200 dilution, ZB-2301; ZSGB-BIO, Beijing, China) and reacted with a 3,3′-diaminobenzidine (DAB) kit (ZLI-0931, ZSGB-BIO) for the color reaction. The Hip and NAc sections were examined with an Olympus Fluo View 1200 confocal microscope system (Olympus Corp., Tokyo, Japan), and photomicrographs of representative Hip and NAc areas were obtained.

### Western blot assay

The western blot assay was conducted as previously described (28). The Hip and NAc were rapidly dissected, and the proteins were extracted with radioimmunoprecipitation (RIPA) buffer (P0013B; Beyotime, Shanghai, China) containing a protease inhibitor cocktail (ST506; Beyotime) on ice. The protein concentration of the supernatant fraction was determined using a bicinchoninic acid (BCA) assay (P0012; Beyotime). Sample proteins were subjected to 10% sodium dodecyl sulfate-polyacrylamide gel electrophoresis (SDS-PAGE). Then, the proteins were transferred to a polyvinylidene fluoride (PVDF) membrane using the Trans-Blot wet transfer system. The PVDF membrane was incubated in a blocking solution at room temperature. The PVDF membrane was probed overnight at 4°C using anti-rat HCN1 monoclonal antibody (1:1,000) and anti-BDNF (1:1,000). The next day, the membrane was washed three times, 10 min each time, with PBS-T and incubated at room temperature with secondary antibodies, including anti-HCN1 [Peroxidase-Conjugated Affinipure Goat Anti-mouse IgG (H + L), 1:200] and anti-BDNF [Peroxidase-Conjugated Affinipure Goat Anti-Rabbit IgG (H + L), 1:200], and reacted with the Immobilon western chemiluminescent HRP substrate (WBKLS0500; Millipore, Bedford, MA, USA) for the color reaction. Then, the gray values of the immunoreactivity were quantified with ImageJ software.

### Quantitative real-time polymerase chain reaction

The rats were euthanized after treatment, and the Hip and NAc were extracted from each group and analyzed. Total RNA was isolated using TRIzol reagent (Cat# 15596–026, Invitrogen, Carlsbad, CA, USA). The RNA concentration and purity (OD260/280) were determined using a NanoDrop ND1000 spectrophotometer (NanoDrop Technologies, Inc., Wilmington, DE, USA). First-strand cDNA synthesis was performed using the RevertAid™ First Strand cDNA Synthesis Kit (Code No. RR047A; TaKaRa, Ohtsu, Japan) in a 20-μL reaction volume with 1 μg template RNA. The PCR reaction mix was prepared according to the manufacturer's protocol. The PCR primer sequences are shown in Table 1. The amplification reactions were performed in 96-well plates using the 7900HT Fast Real-Time PCR System (Cat# 4346906; Applied Biosystems, Foster City, CA, USA). The thermocycling conditions were set according to the manufacturer's protocol. The specificity of amplification was confirmed using a melting curve analysis. Differential gene expression between the drug and saline groups was calculated using the 2^−ΔΔCT^ method with β-actin as an endogenous control.

**Table 1:** The PCR primer sequences designed by Primer Premier 5.0.

**Gene**	**Primer sequence(5′-3′)**	
	**Forward**	**Reverse**
BDNF	CAGCGCGAATGTGTTAGTGGTTA	CAGTGGACAGCCACTTTGTTTCA
HCN1	CTGGGATGGCTGTCTTCAGTTTC	GCGCCAGTAACCAATGCAC
β-actin	GGAGATTACTGCCCTGGCTCCTA	GACTCATCGTACTCCTGCTTGCTG

### Transmission electron microscopy

The TEM test was carried out as described by Marcelo (29). The Hip and NAc tissues were immediately separated, and immersion fixation was completed at ~1 mm3 size. The samples were rinsed in cold PBS and placed in 2.5% glutaraldehyde. The samples were post-fixed with 1% aqueous osmium tetroxide in 0.2 M cacodylate buffer for 2 h. Then, the tissue was rinsed with distilled water before dehydration in a gradually increasing ethanol series and infiltrated using a mixture of half acetone and half resin overnight at 4°C. Following post-fixation in osmium tetroxide and dehydration in an ascending ethanol series followed by propylene oxide, the samples were embedded in Araldite resin (Agar Scientific, Essex, UK) overnight. The 70-nm-thick ultrathin sections were stained with 3% uranyl acetate for 20 min and 0.5% lead citrate for 5 min and were observed with the JEOL 1200EX at 120 kV (JEOL, Inc., Tokyo, Japan). Ultrastructural changes in Hip and NAc synapses were observed under TEM (HT7700-SS, Hitachi, Tokyo, Japan). Bouton parameters, including synaptic cleft width, postsynaptic density (PSD), and curvature of the synaptic interface, were quantified using ImageJ, in accordance with previous methods (30, 31).

### Statistical analysis

All measurements were obtained by an independent investigator blinded to the experimental conditions. Total ethanol withdrawal scores in the different groups were compared using the Mann–Whitney *U*-test. Between-group differences in body weight were analyzed using repeated-measures analysis of variance (ANOVA). Other data are expressed as the mean ± standard error. Student's *t*-test was used to compare the differences between the control and ethanol groups using SPSS 22.0 software (SPSS Inc., Chicago, IL, USA). The correlation between the HCN1 protein and BDNF mRNA as well as the BDNF protein was tested by correlation analysis. A *P* < 0.05 was considered significant.

## Results

### Determination of the ethanol effect

**Figure 2:**
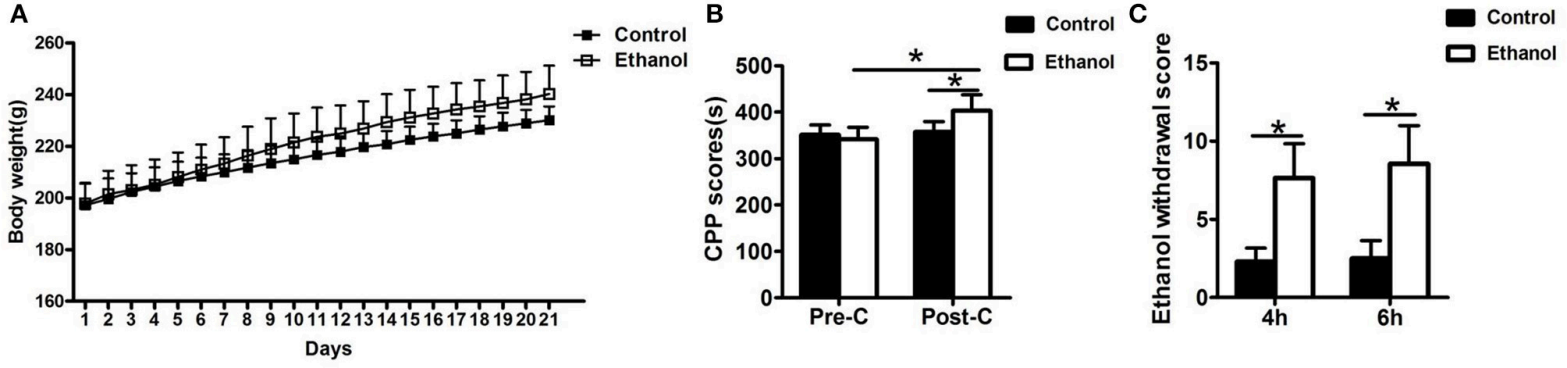
Effects of ethanol dependence on body weight, conditioned place preference (CPP) and total ethanol withdrawal score at hours 4 and 6 of ethanol withdrawal. **(A)** The Ethanol group gained significantly more weight than the group; **(B)** The group exhibited no difference in the Pre-CPP; **(C)** The total ethanol withdrawal score. Data are as mean ± standard error. ^*^P < 0.05 was significant.

The rats in the ethanol group gained significantly more weight than rats in the Control group (*F* = 25819.874, *P* < 0.001; Figure 2A). After the 21-day ethanol administration procedure, the weight of rats in the ethanol group (240.29 ± 10.96 g) increased by 28.87% compared with that of rats in the control group (230.14 ± 5.43 g).

No significant difference was observed in the time spent between the water-paired and ethanol-paired sides in the pre-CPP between the Control and Ethanol groups (*t* = 1.372, *P* > 0.05; Figure 2B). The time spent in the ethanol-paired compartment by the ethanol group was significantly higher during the post-CPP than that of the Control group (*t* = 5.007, *P* < 0.001; Figure 2B).

### Ethanol withdrawal symptoms

#### Ethanol withdrawal score

The total ethanol withdrawal score in the Control group was significantly lower than that in the Ethanol group after 4 h of ethanol withdrawal (*U* = 400, *P* < 0.001; Figure 2C) and after 6 h of ethanol withdrawal (*U* = 392.5, *P* < 0.001; Figure 2C).

#### Ethanol withdrawal anxiety behaviors

**Figure 3:**
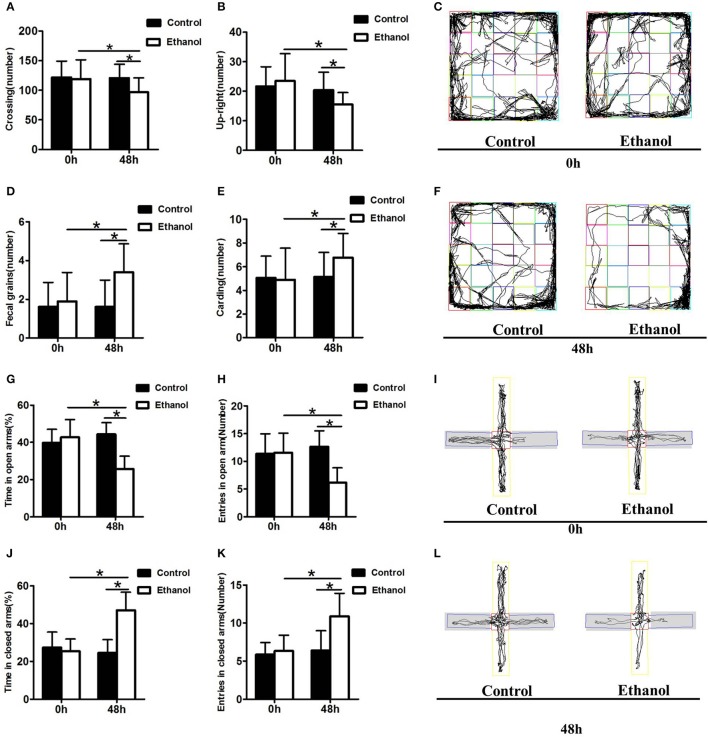
Effect of ethanol withdrawal on the anxiety behaviors. **(A)** The number of crossings in the OFT. **(B)** The number of up-right in the OFT. **(D)** The number of fecal grains in the OFT. **(E)** The number of carding in the OFT. **(C,F)** Representative video tracking images during the last 5 min in the OFT. **(J,G)** Time spent in closed arms and open arms in the EPM. **(K,H)** Number of entries into closed arms and open arms in the EPM. **(I,L)** Representative video tracking images during the last 5 min in the EPM. 0 h: Before ethanol withdrawal period. 48 h: After ethanol withdrawal period. Data are mean ± standard error. ^*^P < 0.05 was significant.

The effect of the ethanol administration procedure on anxiety-like behaviors was shown by the OFT (Figures 3C,F). The number of cardings, fecal grains, crossings, and upright postures did not decrease in the Ethanol group compared with those in the Control group before withdrawal (0 h; carding: *t* = 0.206, *P* > 0.05; Figure 3E; fecal grains: *t* = 0.686, *P* > 0.05; Figure 3D; crossing: *t* = 0.294, *P* > 0.05; Figure 3A; up-right posture: *t* = 0.730, *P* > 0.05; Figure 3B). However, after 48 h of ethanol withdrawal, the rats in the Ethanol group exhibited more anxious behavior, including adding to the number of fecal grains and carding, as well as decrease in the number of crossings and up-right postures compared to those before withdrawal (48 h; carding: *t* = 2.390, *P* < 0.05; Figure 3E; fecal grains: *t* = 3.130, *P* < 0.001; Figure 3D; crossing: *t* = 4.001, *P* < 0.001; Figure 3A; upright posture: *t* = 2.789, *P* < 0.05; Figure 3B). The rats in the Ethanol group exhibited more anxious behavior after ethanol withdrawal, compared with that in the Control group (carding: *t* = 2.448, *P* < 0.05; Figure 3A; fecal grains: *t* = 3.983, *P* < 0.001; Figure 3D; crossing: *t* = 3.189, *P* < 0.05; Figure 3E; upright posture: *t* = 2.957, *P* < 0.05; Figure 3B).

The effect of the ethanol administration procedure on different anxiety-like behaviors was shown by the EPM test (Figures 3I,L), revealing that rats in the Ethanol group exhibited significantly more time in the closed arms (*t* = 8.532, *P* < 0.001; Figure 3J), and significantly more entrances into the closed arms (*t* = 3.731, *P* < 0.05; Figure 3K) than rats in the Control group. The time spent in the open arms (*t* = 2.734, *P* < 0.05; Figure 3G) and the number of entrances into the open arms by the Ethanol group (*t* = 3.900, *P* < 0.05; Figure 3H) markedly decreased compared with those in the Control group after ethanol withdrawal. The rats in the Ethanol group showed anxiety-like behaviors after ethanol withdrawal (time spent in closed arms: *t* = 5.789, *P* < 0.001; Figure 3J; number of entries into closed arms: *t* = 4.368, *P* < 0.001; Figure 3K; time spent in open arms: *t* = 5.889, *P* < 0.001; Figure 3G; number of entries in open arms: *t* = 5.756, *P* < 0.001; Figure 3H).

### Changes in HCN1 and BDNF levels in the NAc and Hip after ethanol withdrawal

**Figure 4:**
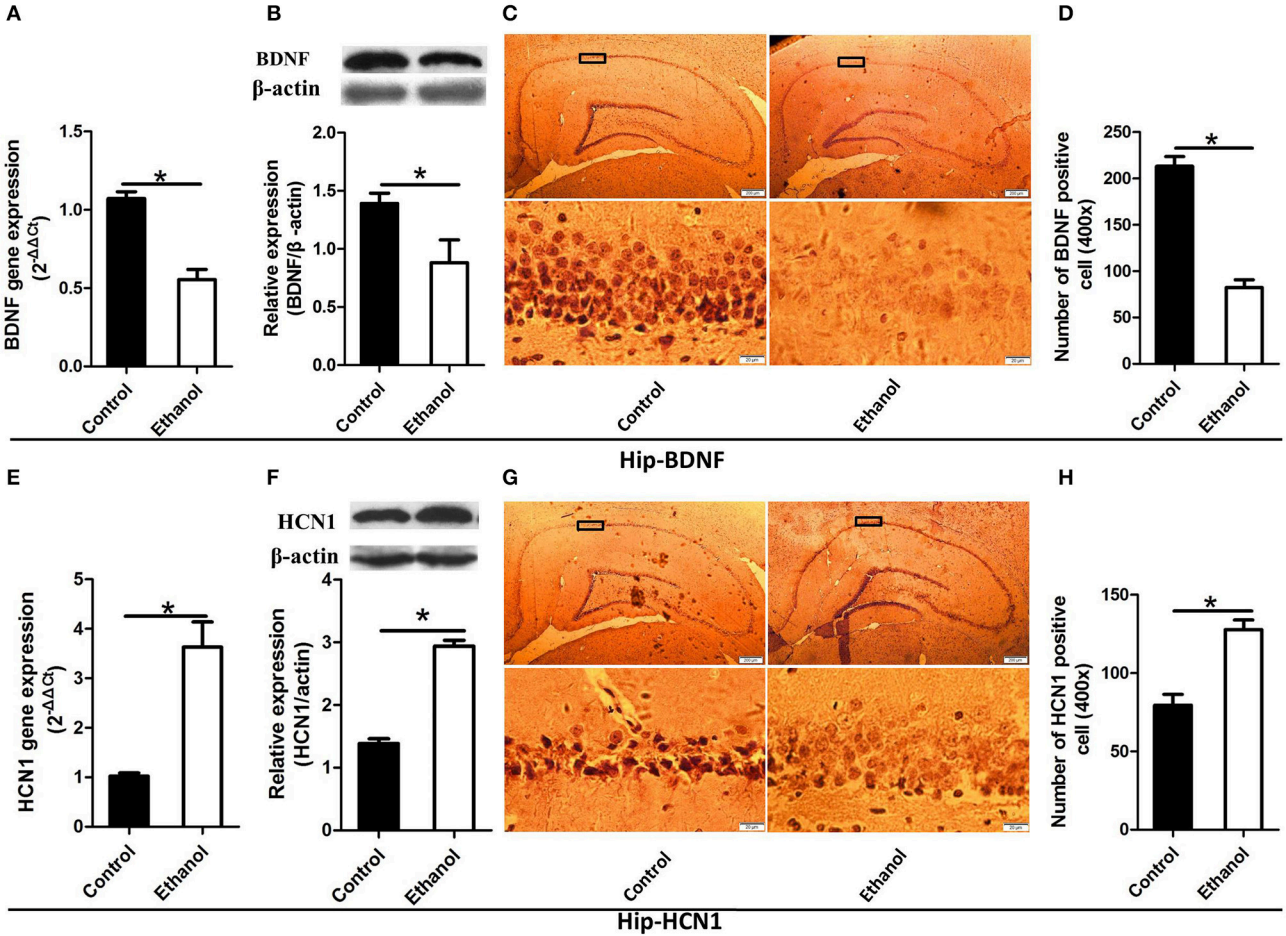
Effects of the ethanol withdrawal procedure on the hyperpolarization-activated cyclic nucleotide-gated cation channel (HCN1) and brain-derived neurotrophic factor (BDNF) protein or gene level changes in the hippocampus (Hip). **(A)** The expression of BDNF BDNF mRNA in the Hip. **(B)** The expression of BDNF-positive cells in the Hip. **(C)** Expression of BDNF positive cells in the Hip. **(D)** The number of BDNF-positive cells in the Hip. **(E)** The expression of HCN1 mRNA in the Hip. **(F)** The expression of HCN1-positive cells in the Hip. **(G)** Expression of HCN1 positive cells in Hip. **(H)** The number of HCN1-positive cells in the Hip. The BDNF- and HCN1-positive cells in the Hip are represented by photomicrographs (400×). Data are mean ± standard error. ^*^P < 0.05 was significant.

**Figure 5:**
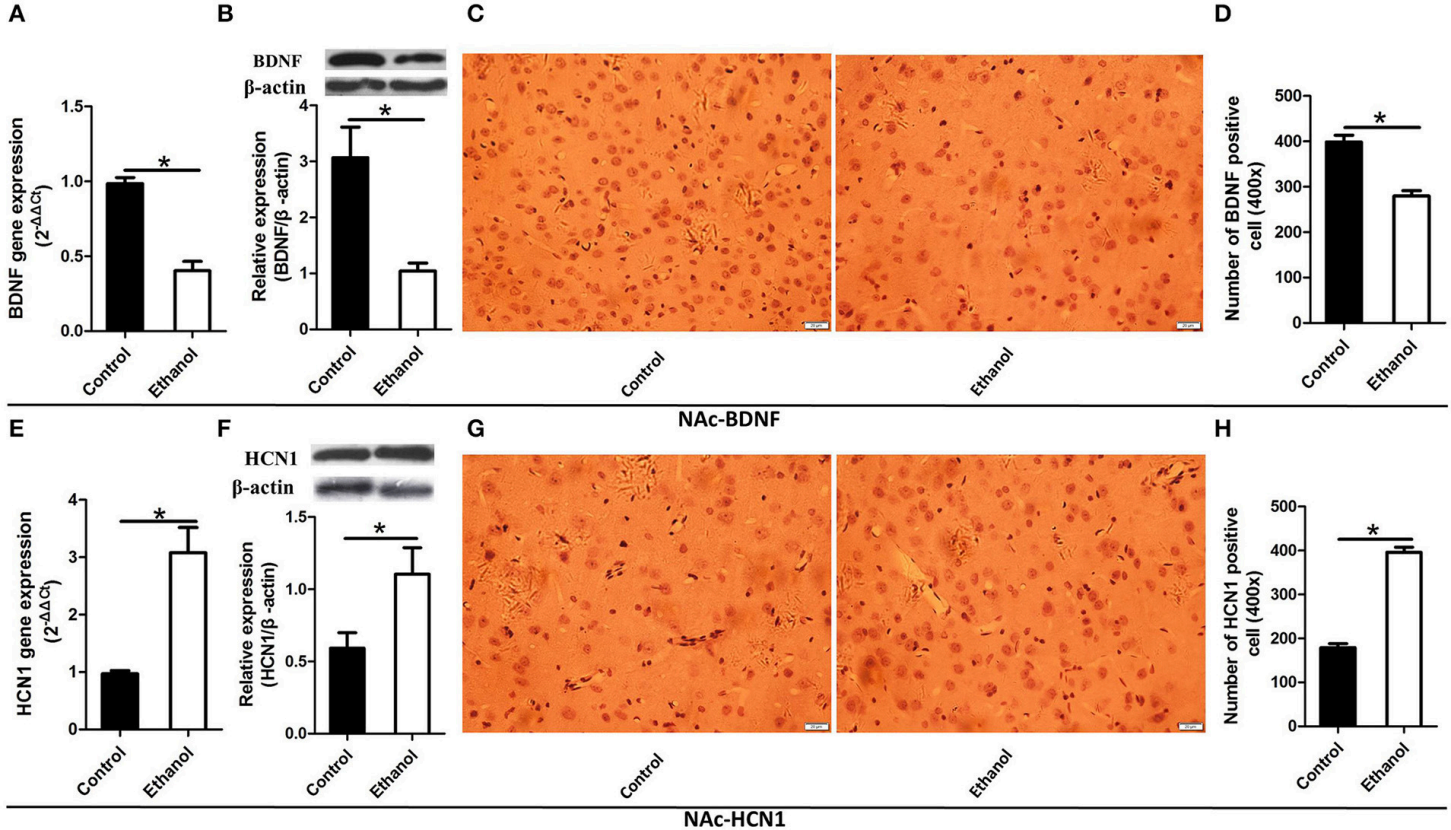
Effects of the ethanol withdrawal procedure on the hyperpolarization-activated cyclic nucleotide-gated cation channel (HCN1) and brain-derived neurotrophic factor (BDNF) protein or gene level changes in the nucleus accumbens (NAc). **(A)** The expression of BDNF BDNF mRNA in the NAc. **(B)** The expression of BDNF-positive cells in the NAc. **(C)** Expression of BDNF positive cells in the NAc. **(D)** The number of BDNF-positive cells in the Hip. **(E)** The expression of HCN1 mRNA in the NAc. **(F)** The expression of HCN1-positive cells in the NAc. **(G)** Expression of HCN1 positive cells in the NAc. **(H)** The number of HCN1-positive cells in the NAc. The BDNF- and HCN1-positive cells in the NAc are represented by photomicrographs (400×). Data are mean ± standard error. ^*^P < 0.05 was significant.

As illustrated in Figures 4, 5, significant decreases in expression of the BDNF gene were detected in the Hip (*t* = 6.541, *P* < 0.001; Figure 4A) and NAc (*t* = 7.791, *P* < 0.001; Figure 5A) following ethanol withdrawal. However, HCN1 expression significantly increased in the Hip (*t* = 5.108, *P* < 0.05; Figure 4E) and NAc (*t* = 4.768, *P* < 0.05; Figure 5E) of the ethanol group.

Because BDNF and HCN1 play a key role in ethanol withdrawal-induced anxiety, we performed a western blot analysis of BDNF and HCN1 in the Hip and NAc. As shown in Figures 4, 5, BDNF expression in the Hip (*t* = 2.360, *P* < 0.05; Figure 4B) was significantly higher in the Control group than that in the Ethanol group and NAc (*t* = 3.585, *P* < 0.001; Figure 5B). The HCN1 level significantly increased in the Hip (*t* = 12.909, *P* < 0.001; Figure 4F) and NAc (*t* = 2.416, *P* < 0.001; Figure 5F) of the ethanol group compared to that in the control group. Through correlation analysis, it was found that there was a negative correlation between the expression of the BDNF gene and the level of the HCN1 protein in the Hip (*r*^2^ = 0.7702, *P* < 0.001) as well as the NAc (*r*^2^ = 0.4747, *P* < 0.001). However, the level of the HCN1 protein had no correlation with the BDNF protein in the Hip (*r*^2^ = 0.3499, *P* > 0.05) or the NAc (*r*^2^ = 0.05422, *P* > 0.05).

The immunohistochemistry results of the ethanol group showed significantly fewer BDNF-positive cells in the Hip (*t* = 9.776, *P* < 0.001, Figures 4C,D) and NAc (*t* = 6.169, *P* < 0.001; Figures 5C,D) compared to those in the Control group. The ethanol withdrawal procedure significantly increased the numbers of HCN1-positive cells in the Hip (*t* = 5.225, *P* < 0.001; Figures 4G,H) and the NAc (*t* = 14.618, *P* < 0.001; Figures 5G,H).

### Changes in synaptic ultrastructure in the NAc and Hip after ethanol withdrawal

**Figure 6:**
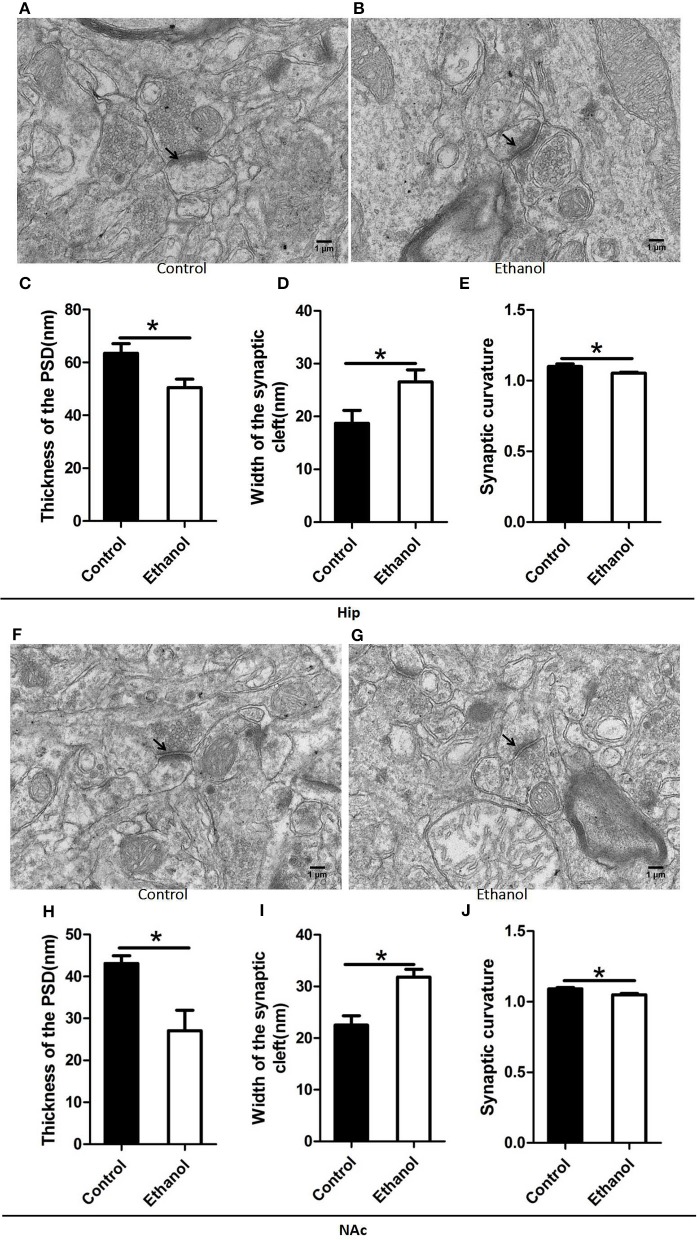
Effects of the ethanol withdrawal procedure on the synaptic ultrastructure in the hippocampus (Hip) and nucleus accumbens (NAc). **(C,H)** Thickness postsynaptic density (PSD) in the Hip and NAc. **(D,I)** Width of the synaptic cleft in the Hip and NAc. **(E,J)** Synaptic curvature in the Hip and NAc. **(A,B,F,G)** Synaptic ultrastructure in the Hip and NAc under ×12,000 magnification. Data are mean ± standard error. ^*^P < 0.05 was significant.

The synaptic ultrastructure of the Hip and NAc was examined by TEM after ethanol withdrawal, as shown in Figure 6. The thickness and curvature of the synaptic interface in the Hip increased in the Ethanol group as compared to those in the control group (*t* = 2.668, *P* < 0.05; Figure 6C; *t* = 2.330, *P* < 0.05; Figure 6E) and NAc (*t* = 3.071, *P* < 0.05; Figure 6H; *t* = 3.845, *P* < 0.05; Figure 6J). In addition, the width of the synaptic cleft was markedly augmented in the Hip (*t* = 0.923, *P* < 0.05; Figure 6D) and NAc (*t* = 0.894, *P* < 0.05; Figure 6I) in the Ethanol group as compared to those in the Control. All the original data of results see in Supplementary Material.

## Discussion

The present study investigated the apparent anxiety behaviors and potential modulation in rats after 48 h of withdrawal from repeated systemic administration of ethanol. The reward effect was assessed by the CPP test, which demonstrated that apparent ethanol addictive behaviors were exhibited in the Ethanol group. As evidenced by the Erden's score results, the rats exhibited ethanol withdrawal symptoms after ethanol was withdrawn. The data for the OFT and EPM tests showed that ethanol withdrawal induced anxiety behaviors. Ethanol withdrawal also increased HCN1 activity and decreased the BDNF level in the Hip and NAc as well as resulted in abnormal changes in synaptic ultrastructure. These results revealed that the Ethanol withdrawal anxious behavior might be based on modulation of synaptic ultrastructure, in which the lowering of BDNF expression down-regulated by higher HCN1 in the Hip and NAc played a potential part.

During the ethanol administration procedure, the body weights of the ethanol group were higher than those of the control, which might have been due to the added calories from ethanol or the positive relationship between ingestion of fat and ethanol, which was hinted at by a previous report (32). However, the developmental trend in body weight observed in the present study differed from that in Rosanne's study, which found that chronic ethanol consumption might cause a thiamine deficiency by inhibiting intestinal absorption of thiamine, leading to weight loss (33), but this difference may have been caused by the different methods of ethanol administration.

The reward properties of ethanol measured by CPP are positively correlated with ethanol consumption (34). The CPP data of the ethanol group suggested that ethanol administration promoted a preference for contextual cues paired with the ethanol experience, which is in accordance with a previous study demonstrating enhanced drug CPP following repeated ethanol consumption (35). However, Tipps reported that contextual association processes are significantly impaired by ethanol intoxication at this dose (36). Considering the changes in body weight and the CPP results, the ethanol-dependent model was established in the present study.

Ethanol withdrawal is known to produce sensitization, tolerance, dependence, craving, and relapse after stopping intake, as shown with other abused drugs (24). The Erden's score of the Ethanol group was significantly higher than that of the Control group, which was interpreted as reflecting the success of ethanol withdrawal.

In the present study, the rats withdrawing from ethanol exhibited a significant decrease in exploratory activity, such as crossing and up-right posturing during the OFT. This decrease in exploratory activity could be interpreted as enhanced anxiety. Furthermore, the evidence of poorer fecal grain and carding performance, which suggests the rat's ability to adapt to a strange environment, implies that the rats were in an anxiety-like state after ethanol withdrawal. These results agree with the study of Bonassoli who also reported that rats exhibit anxious behavior after ethanol withdrawal (22). However, in Bonassoli's study, the rats were habituated to the test apparatus by repeated testing for 3 days, and therefore, the decreased exploration could be considered normal expression rather than confounding of an enhanced anxiety-like state (37).

The EPM has been widely employed to evaluate ethanol withdrawal-induced anxiety-like behavior in rodents (21). Ethanol withdrawal usually decreases exploratory activity on the EPM, indicating the anxiety-like effect of ethanol withdrawal in rodents (37). In the present study, the EPM data from the Ethanol group showed a lower number of entries and percentage of time spent in the open arms compared with those of the control group, which was in accordance with another study describing anxiety-like effects in rodents that were induced with ethanol withdrawal (21). However, Gonzaga reported that the ataxia induced by ethanol also affects exploration during the EPMT (38). Thus, the significant difference in exploration of the closed arms observed in the present study suggesting decreased exploration of the open arms was probably not a result of ethanol withdrawal.

The abnormal changes in the morphological structure of the PSD may lead to disturbances in long-term synaptic plasticity and dysfunction in synaptic transmission (5). In the present study, the PSD revealed reduced thickness in the anxious rats undergoing ethanol withdrawal. Some authors have hypothesized that changes in PSD thickness during synaptic transmission underlie some aspects of the ethanol withdrawal syndrome (22). This may also explain why ionotropic glutamate receptors [iGluRs], which are important constituents of the PSD, show decreased expression after ethanol consumption (39). The iGluRs could disturb the postsynaptic membrane response to the signal by causing intense Ca^2+^ loading (40). In addition, these changes in synaptic ultrastructure might lead to ablation of cyclin-dependent kinase 5, a major regulator of synaptic plasticity, which could also enhance the release of gamma aminobutyric acid and decrease anxiety (41). The crucial role of the PSD thickness in the anxiety-like effects after ethanol withdrawal requires further exploration.

Although some studies have shown that PSD is involved in the postsynaptic membrane response to the signal, few studies have investigated the effects of the synaptic cleft and synaptic curvature as they relate to synaptic functions involved in anxiety after ethanol withdrawal (40). In the present study, we found an increase in synaptic cleft width and a decrease in synaptic curvature in the NAc and CA1 of the Hip after ethanol withdrawal. A potential mechanism of the ethanol withdrawal-induced anxiety by changes in synaptic ultrastructure is that an increase in synaptic cleft width could retard delivery of neurotransmitters from the presynaptic membrane to the postsynaptic membrane (42). Furthermore, the change of synaptic curvature could reflect the functional activity of the neuron. Gondre reported that a curved synapse has more mitochondria than a straight synapse, suggesting that a curved synapse is more active (43). The decreased neuronal activity caused by widening of the synaptic cleft and flattening of the synaptic curvature might be why the rats exhibited anxious behaviors after ethanol withdrawal.

BDNF has been implicated in the development of alcohol addiction due to its role in the regulation of synaptic plasticity in the Hip and NAc (8). BDNF gene expression decreased in rats under ethanol withdrawal, and BDNF signaling and the dendritic spines are involved in anxiety-like behaviors during ethanol withdrawal in rats (44). In addition, treatment with BDNF increases the numbers of dendritic spines and synapses in the Hip (45), suggesting a role for BDNF–Arc signaling in the regulation of neuronal architecture.

In the present study, protein levels of BDNF in the Hip and NAc significantly decreased in the Ethanol group compared to those in the Control group after ethanol withdrawal. Here, BNDF decreased in the NAc and CA1 of the Hip after abstinence from chronic ethanol consumption, suggesting that BDNF is involved in ethanol withdrawal-related anxiety and alcohol-drinking behaviors in rats (9, 10). Additionally, BDNF gene expression also decreased in the NAc and CA1 of the Hip in the Ethanol group. This result contrasts with that of Tapia, who found no difference in BDNF mRNA between the Ethanol withdrawal group and Control group in the CA3 of the Hip 12 h after ethanol withdrawal. The differences in these two studies might be caused by the expression of BDNF mRNA, which was influenced by the different brain regions or the different withdrawal times (46). Some studies show that BDNF signaling promotes the expression of postsynaptic density protein-95 (PSD-95) in synapses and dendritic spines and regulates the post-synaptic localization of PSD-95 via the TrkB signaling pathway (47).

The number of HCN1-positive cells and HCN1 expression levels significantly increased in the Hip and NAc. A more interesting development was that there was a negative correlation between the expression of the BDNF gene and the level of the HCN1 protein, which suggested that level of the HCN1 protein might down regulate the expression of BNDF mRNA. Mala also reviewed the process whereby inhibiting HCN1 channels leads to antidepressant-like effects and enhances the BDNF mRNA level. However, the cellular mechanism (or mechanisms) that reduces HCN1 channel function leading to an increase in BDNF synthesis is unknown (13).

The change in Ih in the HCN1 channel caused by ethanol withdrawal is mediated by increases in cAMP, which binds to a cyclic nucleotide binding domain (CNBD) on the COOH-terminus of the channel. The HCN1 channels are activated by the CNBD (48). However, we found that HCN1 mRNA and protein were expressed, suggesting an unclear mechanism of the ethanol withdrawal effect by the HCN1 gene expression.

## Limitation and outlook

Despite the important role played by Hip and NAc in mediating ethanol withdrawal-induced anxiety, we were unable to ascertain whether or not there is any interaction between Hip and NAc. Additionally, Neuropeptide Y (NPY) is a neuromodulator that is involved in the regulation of alcohol dependence and withdrawal, as well as the BDNF (49, 50). Both ethanol exposure and withdrawal from chronic ethanol consumption has been shown to produce changes in NPY and NPY receptor protein levels. Therefore, whether it plays a critical role in ethanol withdrawal anxiety behaviors has not been observed, and we will continue to explore this important point in future studies.

## Conclusion

In summary, we provide several lines of new evidence for the increase in anxiety-like behaviors exhibited by rats undergoing ethanol withdrawal that possibly resulted from abnormal changes in synaptic ultrastructure. These findings speculate that the extended synaptic ultrastructure might be regulated by HCN1-related BDNF mRNA expression during ethanol withdrawal-induced anxiety, which might lead to a better understanding of the synaptic systems affected by ethanol withdrawal and provide new perspectives for the development of appropriate therapies for ethanol withdrawal-induced anxiety.

## Author contributions

LH, LS, and YG designed the study. LH and YG collected the data. LH analyzed data and drafted the manuscript. BL, YW, CL, GW, QL, JP, HS, and LS reviewed the manuscript. All authors contributed to and have approved the final manuscript.

### Conflict of interest statement

The authors declare that the research was conducted in the absence of any commercial or financial relationships that could be construed as a potential conflict of interest.
